# Non-sedentary Lifestyle Can Reduce Hip Fracture Risk among Older Caucasians Adults: The Adventist Health Study-2

**DOI:** 10.9734/BJMMR/2015/17685

**Published:** 2015-04-27

**Authors:** Vichuda Lousuebsakul-Matthews, Donna Thorpe, Raymond Knutsen, W. Larry Beeson, Gary E. Fraser, Synnove F. Knutsen

**Affiliations:** 1 Center for Nutrition, Healthy Lifestyle and Disease Prevention, School of Public Health, Loma Linda University, Loma Linda, CA, USA; 2 Department of Health Services, Los Angeles, CA, USA; 3 Department of Physical Therapy, School of Allied Health Professions, Loma Linda University, Loma Linda, CA, USA

**Keywords:** Fractures, physical activity, aging, sedentary lifestyle

## Abstract

**Aims:**

The beneficial effect of physical activity on reducing hip fracture risk has been supported in many previous studies. The present cohort study explores the relationship between total daily physical activity expressed as MET-hour/day and hip fracture risk among men over 50 years of age and postmenopausal women (n=22,836).

**Methodology:**

Associations between self-reported hip fracture incidence and total daily physical activity and selected lifestyle factors were assessed using Cox proportional hazard regression.

**Results:**

In gender-specific multivariable models, total activity above average (≥ 51 MET-hours per day for men, ≥ 48 MET-hours per day for women) compared to those with sedentary lifestyle (< 40 MET-hours per day) reduced the risk of hip fracture by 60% among men (HR=0.40, 95%CI: 0.23–0.70) (Ptrend=0.002) and 48% among women (HR=0.52, 95%CI: 0.32–0.84) (Ptrend=0.01).

**Conclusion:**

Our findings suggest that a moderate level of physical activity and avoiding a sedentary lifestyle can reduce the risk of hip fracture among the elderly.

## 1. INTRODUCTION

The current recommended level of physical activity among adults over 50 years of age is at least 2.5 hours per week of moderate-intensity aerobic physical activity or at least 20 minutes of vigorous physical activity 3 days per week and resistance training two to three times per week [1]. The beneficial effect of physical activity on reducing hip fracture risk has been observed in many previous studies [2–9].

However, most studies did not account for the total daily physical activity, as they omitted activities such as work, transportation or household chores. Among women, the majority of daily physical activity may consist mainly of household work activities. Moayeri et al. [10] had expanded the definition of physical activity to include all daily awake time activity (at home, work, transportation and leisure) expressed in MET-hr. Carlsson et al. [11] assessed the total energy expenditure around the clock and reported that small amounts of physical activity can reduce the mortality rate from a variety of diseases. Little is known of the association between total daily physical activity and the risk of hip fracture. This may be due to the imprecision in defining a threshold of physical inactivity which is detrimental to bone health and is often referred simply as a sedentary life style. However, some studies have reported a non-significant positive association between some sedentary lifestyle indicators and the incidence of hip fractures [4,10,12].

The present study explores the relationship between total daily energy expenditure expressed as MET-hours (the ratio of the work metabolic rate to a standard resting metabolic rate (RMR) of 1.0 kcal.kg^−1^.h^−1^) [13] and hip fracture risk among men over 50 years of age and postmenopausal women.

## 2. MATERIALS AND METHODS

### 2.1 Study Population

Subjects were enrollees in the Adventist Health Study-2 (AHS-2), consisting of Adventists throughout the United States and Canada who completed a comprehensive lifestyle and dietary questionnaire at enrollment [14]. This study was approved by the Loma Linda University Institutional Review Board (IRB).

The study population was limited to Caucasian men and women due to low number of hip fractures among the other races. A total of 58,137 Caucasian men and women aged 30 and above, were enrolled into the study from 2002 to 2007 and 47,154 responded to at least one of the two regularly mailed Biennial Hospital History Surveys. After excluding pre-menopausal women and men younger than 50 years as well as subjects who at baseline self-reported osteoporosis, minor trauma fracture and extreme values of daily caloric intake, a total of 22,836 subjects were available for the present analysis.

### 2.2 Baseline Questionnaire

A 50-page comprehensive lifestyle questionnaire was completed by all subjects at enrollment into the AHS-2. This questionnaire included medical history, demographics, a female reproductive history section, a food frequency questionnaire (FFQ) and a separate section on physical activity. Physical activity was assessed using 15 questionnaire items focusing on the subject’s typical physical activity patterns during the previous twelve months. The questions captured the time (< 20 minutes, 20–39 minutes, 40–59 minutes, 1 – < 2 hours, 2 – < 3 hours, 3 – < 6 hours, ≥ 6 hours per day) spent on five of the six levels of activities such as napping, lying down, moderate activity, vigorous activity, extremely vigorous activity on a usual week day, on Saturday and on Sunday (Table 1). Light activities were intentionally not included in the questionnaire as they are difficult to measure accurately. Instead, the amount of time spent on light activities was estimated using the difference between the total hours of accounted activities (sleeping, napping, lying down, moderate activity, vigorous activity, extremely vigorous activity) and 24 hours. Using the 2011 Compendium of physical activities [13], a MET-hr value for each level of activity was obtained by using the average value of all activities falling in the respective levels. All activities were assigned the MET-hr values of 0.95, 0.95, 1.0, 2.0, 4, 7 and 9, respectively (Table 1).

Three levels of daily physical activity (sedentary, below average to average, above average) were defined. Sedentary level was estimated based on the information from the subjects on the baseline questionnaire regarding average number of hours spending on watching TV (2 hours) and sleeping (7 hours). Thus, sedentary was defined as 39 MET-hr or less (7 hours of sleeping (7*0.95=6.65), 2 hours of TV watching (2*1.3=2.6) and the rest of 15 hours in any light activity (15*2=30). Based on this information, a daily physical activity level of greater than 39 MET-hr was considered as non-sedentary. The average time spent on sleeping, napping, lying down, moderate activity, vigorous activity and extremely vigorous activity is shown in Table 2.

The mean daily MET-hr values were 47 and 50 among post-menopausal women and men, respectively. Among the men, those with daily MET-hours ranging from 40 – 50 were classified in the below average to average group and those with the daily MET-hours of at least 51 were classified into the above average group. Among the post-menopausal women, those with daily MET-hours ranging from 40 – 47 were classified in the below average to average group and those with the daily MET-hours of at least 48 were classified into the above average group.

### 2.3 Confounders

A total of 13,593 men and women who reported either a history of osteoporosis or previous fracture were excluded from the analysis. The exclusion was done in order to eliminate any potential hip fracture cases as a consequence of osteoporosis which may obscure the benefit of non-sedentary lifestyle in reducing hip fracture risk. The lifestyle questionnaire also included information on smoking, estrogen use, demographics and a number of doctor-diagnosed chronic diseases that could affect physical activity.

### 2.4 Outcome Measurement

Approximately every two years after enrollment into the parent study, a “Biennial Hospitalization History” questionnaire (HHQ) was sent to study subjects. Eighty-one percent (n=47,154) of Caucasian subjects responded to either the first Biennial Hospital History Survey (HHQ1) or the third Biennial Hospital History Survey (HHQ3). These two HHQs included questions on any fracture (broken bone) of the hip after enrollment. A total of 251 men and women who answered “yes” to this question were identified as hip fracture cases for our study population. Our database was linked with the National Death Index database and used ICD10- S 72.0–72.2 codes to identify additional hip fracture cases among those who died after enrollment and therefore were unable to return the HHQ. Seventeen additional hip fractures were identified for a total of 268 hip fractures.

### 2.5 Statistical Analysis

Chi-square tests and T-tests were used to determine the statistical significance of the association between hip fractures and selected predictor variables. Cox proportional hazard regressions were used to determine the associations between MET categories (sedentary, below average, above average) and occurrence of hip fracture. Hazard ratios and 95% confidence interval (CI) were calculated with attained age as time variable adjusted for all the above covariates. Left truncation of failure time and time at risk were used to select only ages after subjects joined AHS-2. *P*-value of Chi-square difference of the likelihood ratio test between the full model (with MET-hrs categories) and the reduced model (without MET-hrs categories) were determined. All statistical analyses were performed using SAS 9.3 (SAS Institute, Cary, NC).

## 3. RESULTS

Men reported spending more time at higher intensity activities than women and a larger proportion of men, compared to women, had daily MET-hr higher than the mean (47 for females and 50 for males). Compared to post-menopausal women, men reported significantly more daily time spent napping, and doing vigorous as well as extremely vigorous activities (Table 2). Correspondingly, post-menopausal women reported significantly more daily time spent in light activities. Compared to post-menopausal women, men on the average, reported higher activity level equivalent to approximately 3 more MET-hours per day.

A total of 268 participants of our study population experienced a hip fracture, and of these 54% were post-menopausal women and 46% were men. In both men and women, cases were significantly older than non-cases (Table 3). Cases had lower body weight than non-cases, but this difference was only statistically significant among the post-menopausal women. In both genders, there were no significant differences in the daily caloric intake, daily intake of protein and calcium between cases and non-cases. Compared to non-cases, a larger proportion of male cases reported having one or more comorbidities whereas there was no difference among the female cases and non-cases. Among post-menopausal women, current estrogen users experienced less hip fractures as compared to past/never users (17% versus 22%), however, the association was not statistically significant. In both genders, those who reported being physically active above the sedentary level had a significantly lower incidence of hip fractures.

### 3.1 Univariate Analysis

In both genders, any activities beyond sedentary levels were associated with reduced risk of hip fractures (Tables 4 and 5). Compared to those with sedentary activity, hazard ratios (HR) for hip fracture among men with physical activity equivalent to 40–50 MET-hr (low to average) and >50 MET-hr per day were 0.47 (95% CI: 0.30–0.72) and 0.41 (95% CI: 0.24–0.70), respectively (*P*-trend=0.002). For post-menopausal females, the corresponding values for 40–47 MET-hr and >47 MET-hr per day were 0.57 (95% CI: 0.38–0.85) and 0.63 (95% CI: 0.40–1.00), respectively (*P*-trend=0.07).

### 3.2 Multivariable Analyses

When the analyses were adjusted for weight, height, caloric intake, protein intake, calcium intake, smoking status, estrogen usage (women) and a number of co-morbidities (angina pectoris, myocardial infarction, stroke, transient ischemic attack, congestive heart failure, chronic bronchitis, emphysema, asthma, Parkinson’s disease, cataract, macular degeneration, rheumatoid arthritis, osteoarthritis, degenerative disc, osteoporosis, fibromyalgia, systemic lupus erythematosus, cancer) that could affect physical activity levels, the hazard ratios associated with the various MET-hr levels were strengthened in the highest category in both men and women (0.40 (95% CI: 0.23–0.70) and 0.52 (95% CI: 0.32–0.84)). In both genders, a significant difference in the likelihood ratio test between the full model (with MET categories) and the reduced model (without MET categories) was observed.

## 4. DISCUSSION

Our finding of a significant protective effect of moderate physical activity on hip fracture risk is supported by others. Previous studies have found that physical activity reduced the risk of hip fracture by 30–50% [2–9]. Most studies focused on leisure time physical activities such as sports or weight bearing exercise. In the Women’s Health Initiative (WHI) study, postmenopausal women who had no leisure time physical activity significantly increased their risk of hip fracture by 64% compared to women who engaged in moderate physical activity equivalent to at least 12 MET-hr per week [15]. In the Nurses’ Health study, postmenopausal women who reported engaging in leisure time physical activities equivalent to at least 9 MET-hr per week significantly reduced the risk of hip fracture by 33% compared to those with less than 3 MET-hr per week [4]. In a study among men, compared with the lowest quartile of leisure physical activity (0.5 MET-hr per day) and after adjustment for all covariates, men with MET index quartiles 2 through 4 combined tended to have a lower risk of hip fracture (HR 0.58; 95%CI 0.32–1.03) [16].

Only a few studies have looked at all types of daily physical activity [5,10] and estimated MET-hr values similar to ours [4,10,15,16]. In the EPIC-Norfolk study, physical activity at home, at work, during transportation, at leisure time and total physical activities were estimated in MET-hr per week [10]. Comparing the second quartile of women to the first, physical activity at home above 39.3 MET-hr per week and leisure activity above 11.2 MET-hr/week significant reduced the risk of hip fracture by 49% and 45%, respectively. Among men, total physical activity level above 67.5 MET-hr per week (2^nd^ quartile vs. 1^st^) and leisure activity above 54.9 MET-hr per week (4^th^ quartile vs. 1^st^) significantly reduced the risk of hip fracture by 70% and 88%, respectively.

Our finding suggests that any moderate form of physical activity compared to being sedentary (Tables 4 and 5) reduces the risk of hip fracture in both men and post-menopausal women by 60% and 48%, respectively. The benefit of increased mobility rather than sedentary habits was also observed in a prospective study of 9,704 older nonblack women followed for an average of 7 years. Women who engaged in household chores such as yard work, gardening, sweeping, etc. for more than 9 hours per week reduced their risk of hip fracture by 22% compared to those with less than 5 hours per week [5]. Furthermore, those women who reported sitting more than 8 hours per day increased their hip fracture risk by 37% compared to those sitting less than 6 hours. Another prospective study among postmenopausal Caucasian women found that sitting more than 4 hours per day, compared to less than 4 hours per day, increased the risk of hip fracture by 70% [17]. The Nurses’ Health study reported that standing for more than 40–54 hours per week lowered the risk of hip fracture by 34% [4]. The EPIC-Norfolk study found that housework activity of at least 25 MET-hr/wk among women reduced the risk of hip fracture by 66% [10].

Our findings showed that among postmenopausal women, the benefit of physical activity on hip fracture risk reached a threshold at physical activity levels of below average to average levels (40 – 47 MET-hr per day), indicating that activity beyond the average did not reduce their hip fracture risk further. The threshold effect observed among women in our study was not observed among men. The more active these men were, the lower their risk of hip fracture. One explanation for this finding could be the difference in the intensity of the daily activities among men compared to women. With the same MET-hr values, men may spend more time on vigorous and extremely vigorous activities per day. In our study, the average time spent on any vigorous activity among men was 0.83 hr per day compared to 0.59 hr per day among women (Table 2). In addition, the average time spent on extremely vigorous activity among men and women was 0.45 hr per day and 0.12 hr per day, respectively (Table 2).

Another explanation could be the difference in muscle mass between men and women at the same age. Elderly women seem to lose muscle mass faster compared to men at the same age [18]. It is also possible that women may have weaker bones with lower bone mineral density (BMD). Previous studies have reported gender differences in the effect of physical activity on fracture risk where physical activity in younger years decreased the risk of fractures in older postmenopausal women, but not in men [19,20]. This suggests that the mechanical stimulation of bone growth prior to the period of rapid bone loss after menopause could determine the beneficial effect of physical activity among women [19,21]. Physical activity above the average level could expose some aging women to higher risk of hip fracture due to weaker bone, at least partly caused by accelerated bone loss due to estrogen depletion after menopause. Men may reap more benefit from exercise in terms of maintaining their BMD compared to women [22,23]. A meta-analysis of randomized controlled trials among postmenopausal women found that ground or joint reaction force exercise increased BMD at the femoral neck by 0.29 g/cm^2^ [22]. A similar meta-analysis among men reported an improvement of BMD at the femoral neck of 0.58 g/cm^2^ [23].

As there is a difference in BMD and muscle mass between men and women, women are more likely to experience a hip fracture if they fall. Despite numerous studies demonstrating the benefit of vigorous physical activity on reducing hip fractures [2,4–6,15], elderly women should have a proper level of training and conditioning before engaging in vigorous activity. This is supported by several studies showing the benefit of balance training in reducing the risk of fall-related fractures among the elderly [24–26].

Co-morbid conditions may significantly reduce exercise and result in weaker bone and lower muscle mass. Therefore, adjusting for co-morbid conditions was performed in order to differentiate between sedentary lifestyle as behavior and immobility due to illness. The protective effect of physical activity persisted and became stronger indicating the benefit of physical activity despite co-morbidity conditions. Besides co-morbid conditions, some prescribed medications can also increase the risk of hip fracture and limit physical activities. Due to limited information on subjects’ prescribed medication, we were not able to adjust for this factor.

### 4.1 Strengths and limitations

A strength of this study is the prospective design with a follow-up period of at least 2 years. In addition, important confounders were adjusted for in the model including a number of co-morbidities. There are some limitations due to potential measurement error of both exposure and outcome variables. For the outcome variable, non-response bias may exist because some subjects might fail to report their hip fracture status. However, with the questionnaire non-response rate of 19%, we assume that any associated underreporting of hip fracture is not associated with physical activity. In addition, the self-reported hip fracture status in our study could possibly have been improved by verification with hospital records. However, literature has shown that the validity between self-reported hip fracture and hospital records is very high ranging from 81% to 93% [27,28]. Furthermore, higher education increases the accuracy of self-reported fractures [29]. Since 93% of our study population was high school graduates, this probably resulted in a higher proportion of valid self-reported hip fractures in our study population. For the exposure variable, the MET values for daily physical activity are approximate, and may not have accounted for all daily activities (*e.g.,* sexual activity). In addition there might have been changes in physical activity during the period between the baseline questionnaire and the hip fracture. However, since our study has a prospective design, the misclassification of physical activity level is not likely to be associated with the incidence of hip fracture.

## 5. CONCLUSION

This study took into account all physical activities ranging from household chores to sports activity. Our findings suggest that total daily energy expenditure from these activities may be a better indicator of non-sedentary physical activity than questions on leisure time physical activity since old age limits functional ability to engage in outdoor activities or sports programs. Even with a modest level of physical activity, we found that the risk of hip fracture among the elderly was reduced by at least 40%. In fact, any type of physical activity beyond being completely sedentary was protective. Thus, our findings clarify the apparent benefit of any physical activity beyond a sedentary level.

In conclusion, this study suggests that even very modest levels of physical activity decreases the risk of hip fracture among the elderly. Recommendation to maintain mobility and avoiding a sedentary lifestyle during older years seems to be an important non-pharmacologic intervention to reduce hip fracture risk.

## Figures and Tables

**Table 1:** Physical activities questionnaire and MET values^a^

Activity intensity	MET values^a^	Average met values
**Sleeping**	0.95	0.95
**Napping**	0.95	0.95
**Lying down**		1.0
Reading while lying down	1.3	
Watching TV while lying down	1.0	
**Light Intensity activities**		2.0
Cooking	2.5	
Washing dishes	2.5	
Slow walking	2.0	
Driving	2.0	
Hobbies working at a desk or standing still	1.8	
Desk work	1.5	
Watching TV while sitting	1.3	
Hand-watering plant/yard	1.5	
**Moderate intensity activities**		4.0
Swimming leisurely	6.0	
Mowing lawn (power mower)	5.5	
House paint	5.0	
Golfing	4.8	
Fast walking	4.5	
Repeated lifting of objects up to 15 lbs.	4.5	
Casual Cycling	4.0	
Carpentry	4.0	
Calisthenics (moderate)	3.8	
Active child care	3.5	
Gardening	3.5	
Sailing	3.3	
Vacuuming/mopping	3.3	
Cleaning windows	3.2	
Patient care	3.0	
**Vigorous intensity activities**		7.0
Fast Cycling	10.0	
Vigorous lap swimming	9.8	
Calisthenics (vigorous)	8.0	
Tennis	7.3	
Aerobics	7.3	
Skiing	7.0	
Scrubbing floors	6.5	
Moderate run/jog	6.0	
Team sports	6.0	
Repeated lifting of objects up to 20–35 lbs.	6.0	
Hoeing	5.0	
Patient lifting	4.5	
**Extremely vigorous intensity activities**		9.0
Marathon	13.3	
Racquet ball	10.0	
Working with heavy tools	9.0	
Fast running	9.0	
Continuous digging	8.8	
Heavy weight lifting	8.0	
Repeated lifting of objects up to 40 lbs. or more	8.0	
Carrying 40 lbs. or more	8.0	
Digging	7.8	
Chopping with heavy tools	6.3	

a 2011 Compendium of Physical Activities: A Second Update of Codes and MET Values [13]

**Table 2:** Time spent on physical activities reported among 22,836 caucasian men and Post-menopausal Women

	Post-menopausal women		Men (n=10,597)		P value

	Hrs/day mean (SD)^a^	MET-hr	Hrs/day mean (SD)^a^	MET-hr	
Sleep hours per day	7.1 (1.1)	6.7	7.2 (1.0)	6.8	0.03
Nap hours per day	0.3 (0.4)	0.3	0.4 (0.5)	0.4	<0.001
Sedentary activity per day	0.5 (0.9)	0.5	0.5 (0.9)	0.5	0.35
Light activity per day	13.5 (3.1)	27	12.8 (3.7)	25.6	<0.001
Moderate activity per day	1.8 (1.8)	7.2	1.8 (1.8)	7.2	0.81
Vigorous activity per day	0.59 (1.0)	4.1	0.83 (1.3)	5.8	<0.001
Extremely vigorous activity per day	0.12 (0.5)	1.1	0.45 (1.0)	4.0	<0.001
Average daily MET-hr		46.9		50.3	

a SD, Standard deviation

**Table 3:** Demographic and lifestyle characteristics among 22,836 caucasian men and post-menopausal women

	Post-menopausal women			Men		

	Non-cases (n=12,093)	Hip fracture cases (n=146)	P value	Non-cases (n=10,475)	Hip fracture cases (n=122)	*P* value
**Age (years ), mean** (SD)^a^	63.2 (10.9)	74.7 (10.2)	<0.0001	66.1 (10.3)	75.4 (9.6)	<0.001
**Weight (kg ), mean** (SD)^a^	72.9(17.4)	66.8(14.6)	<0.0001	84.3 (15.8)	81.4 (16.4)	0.06
**Height (cm ), mean** (SD)^a^	163.1 (7.0)	162.7(6.9)	0.50	177.7 (7.3)	177.8 (8.4)	0.81
**Energy intake (calories), mean** (SD) a	1699.8 (687.7)	1766.0 (725.4)	0.25	1882.2 (733.0)	1866.3 (760.3)	0.81
**Total daily protein intake (g), mean** (SD) a	61.3 (26.8)	62.2 (36.1)	0.67	66.6 (28.3)	61.9 (27.0)	0.07
**Total daily calcium intake (mg), mean** (SD) a	1103.0 (588.7)	1051.5 (540.0)	0.29	882.8 (500.0)	907.3 (533.3)	0.59
**Estrogen usage (Females):**						
Past/never Users	78.1%	82.9%	0.17			
Current Users	21.9%	17.1%				
**Smoking status**						
Never smokers	84.5%	91.8%	0.02	73.1%	73.8%	0.86
Ever smokers	15.5%	8.2%		26.9%	26.2	
**Co-morbidity** b						
No	62.3%	56.2%	0.13	63.2%	50.8%	0.005
Yes	37.7%	43.8%		36.8%	49.2%	
**Daily metabolic Values**						
< 40 MET-hrs	13.1%	27.4%	<0.0001	10.7%	26.2%	<0.001
Females: 40 – 47 MET-hrs; Males: 40–50 MET-hrs	56.6%	47.3%		59.3%	52.5%	
Females: 48+ MET-hrs Males: 51+ MET-hrs	30.3%	25.3%		30.0%	21.3%	

a SD, Standard deviation

b angina pectoris, myocardial infarction, stroke, transient ischemic attack, congestive heart failure, chronic bronchitis, emphysema, asthma, Parkinson’s disease, cataract, macular degeneration, rheumatoid arthritis, osteoarthritis, degenerative disc, osteoporosis, fibromyalgia, systemic lupus erythematosus, cancer

**Table 4:** Associations between MET-hr categories and Hip fracture incidence among caucasian men^a^

	Univariate analysis (non-cases=10,475; cases = 122)		Multivariate analysis a (non-cases=10,475; cases = 122)^b^	

Daily metabolic expenditure values	Hazard ratio	95% CI	Hazard ratio	95% CI
< 40 MET-hrs (Sedentary)	1.00	Reference	1.00	Reference
40–50 MET-hrs (Below to average)	0.47	(0.30–0.72)	0.44	(0.28–0.69)
51+ MET-hrs (Above Average)	0.41	(0.24–0.70)	0.40	(0.23–0.70)
*P trend=0.002*			*P trend=0.002*	*P*^c^ *=0.001*

a Adjusted for Age, weight, height, Caloric Intake, Smoking, Total Calcium Intake, Total Protein Intake, Co-morbidity Conditions (angina pectoris, myocardial infarction, stroke, TIA, CHF, chronic bronchitis, emphysema, asthma, Parkinson’s disease, cataract, macular degeneration, rheumatoid arthritis, degenerative arthritis, degenerative disc, osteoporosis, fibromyalgia, SLE, cancer)

b 30 subjects were excluded from the model due to censored and missing values

c P value of Chi-square difference of the likelihood ratio test between the full model (with MET categories) and the reduced model (without MET categories)

**Table 5:** Associations between MET-hr categories and Hip fracture incidence among caucasian post-menopausal women^a^

	Univariate analysis (non-cases=12,093; cases = 146)^b^		Multivariate analysis^a^ (non-cases=12,093; cases = 146)^c^	

Daily metabolic expenditure values	Hazard ratio	95% CI	Hazard ratio	95% CI
< 40 MET-hrs (Sedentary)	1.00	Reference	1.00	Reference
40 – 47 MET-hrs (Below to average)	0.57	(0.38–0.85)	0.52	(0.35–0.78)
48+ MET-hrs (Above Average)	0.63	(0.40–1.00)	0.52	(0.32–0.84)
*P trend=0.07*			*P trend=0.0*	*P*^d^*=0.007*

a Adjusted for Age, weight, height, Caloric Intake, Smoking, Total Calcium Intake, Total Protein Intake, Estrogen Usage, Co-morbidity Conditions (angina pectoris, myocardial infarction, stroke, TIA, CHF, chronic bronchitis, emphysema, asthma, Parkinson’s disease, cataract, macular degeneration, rheumatoid arthritis, degenerative arthritis, degenerative disc, osteoporosis, fibromyalgia, SLE, cancer)

b 4 subjects were excluded from the model due to missing values

c 318 subjects were excluded from the model due to censored and missing values

d P value of Chi-square difference of the likelihood ratio test between the full model (with MET categories) and the reduced model (without MET categories)
